# *DNMT1* and *DNMT3A* haplotypes associated with noise-induced hearing loss in Chinese workers

**DOI:** 10.1038/s41598-018-29648-4

**Published:** 2018-08-15

**Authors:** Enmin Ding, Jing Liu, Haoran Guo, Huanxi Shen, Hengdong Zhang, Wei Gong, Haiyan Song, Baoli Zhu

**Affiliations:** 1 Institute of Occupational Disease Prevention, Jiangsu Provincial Center for Disease Prevention and Control, Nanjing, Jiangsu Province China; 2 Nanjing Prevention and Treatment Center for Occupational Disease, Nanjing, Jiangsu Province China; 3 School of Public Health, Southeast University, Nanjing, Jiangsu Province China; 4 Kunshan Centers for Disease Prevention and Control, Kunshan, Jiangsu Province China

## Abstract

This study was conducted to explore the effects of *DNMT1* and *DNMT3A* polymorphisms on susceptibility to noise-induced hearing loss (NIHL) in Chinese workers. A total of 2689 industrial workers from a single textile factory were recruited. Venous blood was collected, as were questionnaire and pure-tone audiometry (PTA) data by specialist physicians. Four selected SNPs (rs7578575, rs749131, rs1550117, and rs2228611) in *DNMT1* and *DNMT3A* were genotyped in 527 NIHL patients and 527 controls. Then, main effects of the genotypes and their interactions were evaluated. Results revealed that the GG genotype at rs749131 and the AG/GG genotypes at rs1550117 and rs2228611 [odds ratio (OR) = 1.87, 2.57, and 1.98 respectively], as well as the haplotypes AGGG and TGGA (rs7578578-rs749131-rs1550117-rs2228611) (OR = 1.35 and 1.56, respectively) were associated with an increased risk of NIHL in the Chinese population. Multifactor dimensionality reduction analysis indicated that rs7578575, rs749131, and rs2228611 interact and are related to increased NIHL risk (OR = 1.63). The genetic polymorphisms rs749131 G, rs1550117 G, and rs2228611 G within the *DNMT1* and *DNMT3A* genes are associated with an increased risk of NIHL in the Chinese population and have the potential to act as biomarkers for noise-exposed workers.

## Introduction

Occupational noise is one of the most common occupational hazards affecting the health of industrial workers, and noise-induced hearing loss (NIHL) is the second most frequent form of sensorineural hearing impairment besides age-related hearing loss (ARHL) worldwide^1^. NIHL is a multifactorial disease resulting from the interaction of genetic and environmental factors^2^. Currently, the mechanism of NIHL is not completely understood. The possible etiopathogenesis may involve inner ear cell apoptosis or necrosis caused by oxidative stress or metabolic products generated during signal transduction and direct mechanical injury to the structures of the cochlea^3–5^. However, numerous population studies have indicated that individuals exhibit various degrees of NIHL risk, even when they are exposed to equal noise intensity levels^1,6^. Animal experiments also have demonstrated that genetic variants contribute to the incidence of NIHL^7,8^. Previous studies have found that single nucleotide polymorphisms (SNPs) in the *HSP70*, *EYA4*, *CDH23, GRHL2*, and *DFNA5* genes are associated with genetic susceptibility to NIHL in humans and may increase or decrease the risk of NIHL through interactions with occupational noise^9–11^. The prevailing evidence indicates that genetic susceptibility and interactions with environmental factors may play an important role in the occurrence and development of NIHL.

DNA methylation represses gene expression through the allosteric inhibition of transcription factors and the transcriptional machinery while serving as a recognition site for a host of repressive factors^12^. This modification is applied by DNA methyltransferase (*DNMT*) proteins. DNA methyltransferase 1 (*DNMT1*) is a maintenance methyltransferase, while *DNMT3A* and *DNMT3B* are *de novo* methyltransferases^13,14^. DNA methylation is recognized as an essential epigenetic mark that regulates chromatin structure and gene expression throughout the genome. Recently, hereditary sensory neuropathy with dementia and hearing loss (HSAN1E) has been shown to be caused by mutations in the maintenance methyltransferase *DNMT1*^15^. Moreover, a family has been identified with adult-onset autosomal dominant cerebellar ataxia with deafness and narcolepsy (ADCA-DN) caused by mutations in *DNMT1*, and the DNA methylation profiles of these individuals have been assessed^16^. An epigenome-wide DNA methylation study investigating the association between hearing ability with age and DNA methylation identified strong associations with DNA methylation in the promoters of 10 genes in humans^17^. Furthermore, oxidative stress has been shown to be associated with DNA hypermethylation, which is mediated by the deregulation of epigenetic-related enzymes such as *DNMT3A*, *H2AFZ*, and *H3F3B*^18^.

Associations between NIHL and *DNMT* SNPs and their functional significance in the *DNMT3A* genes have not yet been reported and previous study by Hu *et al*. showed no significant association between three *DNMT1* SNPs (rs12984523, rs16999593, and rs2228612) and NIHL susceptibility in the Chinese population^19^. However, considering the vital roles of *DNMT1* in hearing disorders and of *DNMT3A* in oxidative stress, we speculated that polymorphisms in *DNMT1*, *DNMT3A* and their interactions could be associated with genetic susceptibility to NIHL. Therefore, we performed a case-control study to elucidate the associations between four *DNMT1* and *DNMT3A* SNPs, namely rs7578575, rs749131, rs1550117, and rs2228611, and genetic susceptibility to NIHL.

## Materials and Methods

### Subjects

This research has been approved by the Jiangsu Provincial Center for Disease Prevention and Control ethics committee (Nanjing, Jiangsu province, China) and all the experiments were carried out in accordance with relevant guidelines and regulations. Informed consent was obtained from each participant before beginning the study. Industrial employees from a single textile factory in eastern China who received annual health examinations performed by the Jiangsu Provincial Center for Disease Prevention and Control were recruited in 2015. A total of 2689 people participated in the health examinations. Occupational health examination items mainly included venous blood collection, general physical examination, and pure-tone audiometry (PTA). During the health examination, personal medical history, smoking and drinking habits, and habitual use of drugs were assessed. In this study, subjects who had quitted smoking or drinking for at least one year were defined as ever smokers and drinkers. Workers who drank a bottle of beer or fifty grams of wine per day for at least one year were defined as “now drinkers”, and subjects who had one cigarette per day for at least one year were defined as “now smokers”. The following subjects were excluded from this study: workers with diseases that may affect hearing thresholds (e.g. otitis media, cholesteatoma, and ear canal stenosis) and workers who have used or are using ototoxic drugs (e.g. aspirin, quinolones, and aminoglycosides). A total of 2477 subjects meet our criteria and were included.

### PTA and NIHL assessment

PTA was performed for each participant after a break in noise exposure for up to 12 hours. Using a Madsen Voyager 522 audiometer (Madsen, Taastrup, Denmark), audiometry was conducted by qualified doctors in a soundproof room.

### Defining NIHL and control subjects

Hearing loss and normal hearing were defined according to Chinese diagnostic criteria for occupational noise-induced deafness (GBZ 49–2007). In this study, occupational noise exposure was defined as levels of noise exposure (Lex) of at least 85 dB (A) during a nominal 8-hour working day. Hearing loss was identified using binaural hearing thresholds that exceed 25 dB at both high (3000, 4000, 6000 Hz) and speech (500, 1000, 2000 Hz) frequencies. Correspondingly, normal hearing was identified by binaural hearing thresholds below 25 dB both at high and speech frequencies. Hearing thresholds were obtained from the results of PTA. The subjects were divided into two groups: NIHL patients (noise-exposed individuals with hearing loss) and control individuals (noise-exposed individuals with normal hearing). We first selected the NIHL patients, and controls were frequency-matched to the patients by sex, age, and intensity of noise exposure^20^. As a result, 527 NIHL patients and 527 controls were selected from all eligible subjects.

### DNA extraction

Peripheral blood (3 mL) was collected in ethylene diamine tetra acetic acid (EDTA) and used for DNA isolation and genotyping. DNA was extracted from blood samples of subjects using the QIAcube HT and QIAamp 96 DNA QIAcube HT Kit (Qiagen, Dusseldorf, Germany) following the manufacturer’s protocol and was then stored at −20 °C until use.

### SNP selection and genotyping

Target SNPs in the *DNMT1* and *DNMT3A* genes were selected on the basis of data from the 1000 Genomes Project and previous findings from the literature^21^. The criteria for identifying SNPs included a minor allele frequency (MAF) in the Chinese Han population (CHB) >0.10 and a linkage disequilibrium r^2^ value > 0.8. In the end, twelve candidate SNPs were selected using these criteria and Haploview software. Then, we searched PubMed using these candidate SNPs and found that rs7578575, rs749131, and rs1550117 of *DNMT3A* and rs2228611 of *DNMT1* were the most commonly reported; these SNPs were therefore used in the subsequent analysis.

Genotypes for the selected individuals at each polymorphic site were determined using ABI TaqMan SNP genotyping assays (Applied Biosystems, Foster City, CA, USA) and pre-designed commercial genotyping probes and primers. The extracted DNA and genotyping probes and primers were added to TaqMan universal PCR master mix (Roche, Branchburg, NJ, USA) according to the manufacturer’s instructions. Genotyping was then performed on an ABI 7900 real-time PCR system (Applied Biosystems). The results were analyzed using the ABI 7900 system sequence detection software version 1.2.3 (Applied Biosystems).

### Detecting promoter activity of SNP rs1550117

As rs1550117 is located within the 2 kb region upstream of the *DNMT3A* gene, transcription factor binding sites were predicted by TRANSFAC, and a luciferase reporter assay was conducted to detect the effects of SNP rs1550117 on *DNMT3A* promoter activity. The target intron containing the rs1550117 A or G allele was synthesized and then cloned into the *Xho*I/*Hind*III restriction sites of the pGL3-enhancer plasmid (Promega, Madison, WI, USA). The accuracy of the plasmid sequence was confirmed by DNA sequencing. House Ear Institute-Organ of Corti 1 (HEI-OC1) cells were cultured in 24-well plates and transfected with 800 ng of each allele-specific plasmid using Lipofectamine™ 2000 (Invitrogen, Carlsbad, CA, USA). As an internal control, the pRL-SV40 plasmid (Promega) was co-transfected into cells. After 48 h of transfection, dual luciferase activities were determined using a Luciferase Reporter Assay System (Promega). All assays were conducted as three independent replicates.

### Statistical analyses

Statistical analyses were performed using SPSS 23.0 software (Chicago, IL, USA). Goodness-of-fit χ^2^ tests were conducted to determine whether the SNPs were in Hardy-Weinberg equilibrium among the control subjects. Categorical variables are presented as percentages, and continuous variables are presented as the mean ± SD. Odds ratios (ORs) and 95% confidence intervals (95% CI) for genotypes were determined using conditional logistic regression models adjusted for age, sex, smoking, and drinking. Differences in allele-specific promoter activity and gene expression were compared using Student’s *t*-tests or paired *t*-tests. Haplotype analysis was performed using the SHEsis platform^22^. All *P*-values were corrected (*P*_*c*_) using the Bonferroni method, and *P* < 0.05 was used to indicate statistical significance.

## Results

### Demographic characteristics of study subjects and Hardy-Weinberg tests of selected SNPs

The general characteristics (age, sex, smoking, drinking, work time with noise, and noise exposure level) and high-frequency hearing thresholds of the NIHL cases and controls are shown in Table 1. No significant differences were observed between NIHL cases and controls in terms of the general characteristics (*P* > 0.05). However, there was a significant difference between NIHL cases and controls in terms of the high-frequency hearing threshold. The average high-frequency hearing threshold was higher for NIHL patients (35.94 ± 9.96) than for controls (14.09 ± 4.14; *P* < 0.001). General information for the selected SNPs and the results of Hardy-Weinberg tests are shown in Table 2. rs7578575 and rs749131 are located in *DNMT3A* introns, rs1550117 is located in the 2 kb region upstream of *DNMT3A*, and rs2228611 is a synonymous SNP in *DNMT1*. The χ^2^ tests revealed that all selected SNPs were in Hardy-Weinberg equilibrium (*P* > 0.05).

**Table 1:** Demographic characteristics of study subjects.

Variables	Cases (n = 527)		Controls (n = 527)		*P*
	n	%	n	%	
Age (years)					
Mean ± SD	40.61 ± 6.25		40.36 ± 5.94		0.518^a^
≤35	124	23.5	130	24.7	0.877^b^
35–45	296	56.2	295	56.0	
>45	107	20.3	102	19.4	
Sex					
Male	492	93.4	486	92.2	0.475^b^
Female	35	6.6	41	7.8	
Smoking					
Now	307	58.3	293	55.6	0.280^b^
Ever	11	2.1	19	3.6	
Never	209	39.7	215	40.8	
Drinking					
Now	217	41.2	221	41.9	0.870^b^
Ever	10	1.9	12	2.3	
Never	300	56.9	294	55.8	
Work time with noise (years)					
Mean ± SD	18.89 ± 7.55		18.06 ± 7.12		0.068^a^
≤16	225	42.7	242	45.9	0.292^b^
>16	302	57.3	285	54.1	
Expose level with noise (dB)					
Mean ± SD	87.13 ± 7.60		87.37 ± 7.44		0.606^a^
≤85	240	45.5	232	44.0	0.616^b^
85–92	101	19.2	94	17.8	
>92	186	35.3	201	38.1	
High frequency hearing threshold (dB)					
Mean ± SD	35.94 ± 9.96		14.09 ± 4.14		**<0.001** ^a^
≤26	54	10.2	527	100.0	**<0.001** ^b^
>26	473	89.8	0	0.0	

^a^*Students’* t-test.

^b^Two-sided χ^2^ test.

**Table 2:** General information of selected SNPs and Hardy-Weinberg test.

Gene	SNP	Alleles	Chromosome	Functional Consequence	MAF		*P* for HWE^b^
					Control	Database^a^	
*DNMT3A*	rs7578575	A/T	2:25265950	Intron variant	0.242	0.244	0.919
	rs749131	G/T	2:25306755	Intron variant	0.326	0.293	0.099
	rs1550117	A/G	2:25343038	Upstream variant 2KB	0.213	0.220	0.711
*DNMT1*	rs2228611	A/G	19:10156401	Synonymous codon	0.294	0.317	0.256

^a^Data from NCBI dbSNP.

^b^*P* value of Hardy-Weinberg test.

### Multivariate analyses of *DNMT1* and *DNMT3A* SNPs and the risk of NIHL

Four *DNMT1* and *DNMT3A* SNPs were selected for genotyping in the 1054 noise-exposed workers (527 NIHL patients and 527 controls). Table 3 presents the genotype and allele distributions of rs7578575, rs749131, rs1550117, and rs2228611 under the co-dominant, dominant, recessive, and allelic models. Results showed that the genotype frequencies of rs749131, rs1550117, and rs2228611 under the co-dominant model were significantly different between cases and controls (*P* = 0.009, 0.034, and 0.017, respectively). Under the recessive model, the rs749131 GG, rs1550117 AG + GG, and rs2228611 AG + GG genotypes were significantly associated with increased NIHL risk (*P* = 0.002, 0.010, and 0.007, respectively). Subsequent logistic regression analysis to adjust for age, sex, smoking, and drinking showed that individuals with the rs749131 GG, rs1550117 AG + GG, and rs2228611 AG + GG genotypes had increased NIHL risk, with ORs of 1.84 (95% CI = 1.24–2.74), 2.57 (95% CI = 1.22–5.41), and 1.98 (95% CI = 1.19–3.20). Under the allelic model, the rs749131 G (OR = 1.24, 95% CI = 1.03–1.48) allele conferred a significantly increased risk for NIHL (*P* = 0.020). Thus, our data revealed that the *DNMT3A* SNPs rs749131 G and rs1550117 G and the *DNMT1* SNP rs2228611 G may be significantly associated with increased NIHL susceptibility.

**Table 3:** Distribution of four polymorphisms and the association with NIHL.

Gene	Genetic models	Genotypes	Cases		Controls		*P* ^a^	Adjusted OR
			n = 527	%	n = 527	%		(95% CI)^b^
DNMT3A	rs7578575							
	Codominant	AA	36	6.8	36	6.8	0.426	1.00 (Ref.)
		AT	203	38.5	183	34.7		1.11(0.67–1.84)
		TT	288	54.6	308	58.4		0.94(0.57–1.53)
	Dominant	TT	288	54.6	308	58.4	0.214	1.00 (Ref.)
		AA/AT	239	45.4	219	41.6		1.17(0.91–1.49)
	Recessive	AT/TT	491	93.2	491	93.2	1.000	1.00 (Ref.)
		AA	36	6.8	36	6.8		1.00 (0.62–1.62)
	Alleles	T	779	73.9	799	75.8	0.315	1.00 (Ref.)
		A	275	26.1	255	24.2		1.11 (0.91–1.35)
DNMT3A	rs749131							
	Codominant	TT	206	39.1	226	42.9	**0.009**	1.00 (Ref.)
		GT	247	46.9	258	49.0		1.05 (0.81–1.35)
		GG	74	14.0	43	8.2		**1.87 (1.24–2.87)**
	Dominant	TT	206	39.1	226	42.9	0.210	1.00 (Ref.)
		GG/GT	321	60.9	301	57.1		1.17(0.91–1.49)
	Recessive	GT/TT	453	86.0	484	91.8	**0.002**	1.00 (Ref.)
		GG	74	14.0	43	8.2		**1.84(1.24–2.74)**
	Alleles	T	659	62.5	710	67.4	**0.020**	1.00 (Ref.)
		G	395	37.5	344	32.6		**1.24 (1.03–1.48)**
DNMT3A	rs1550117							
	Codominant	AA	10	1.9	25	4.7	**0.034**	1.00 (Ref.)
		AG	175	33.2	175	33.2		**2.49 (1.16–5.35)**
		GG	342	64.9	327	62.0		**2.61 (1.23–5.53)**
	Dominant	AA/AG	185	35.1	200	38.0	0.337	1.00 (Ref.)
		GG	342	64.9	327	62.0		1.13(0.88–1.46)
	Recessive	AA	10	1.9	25	4.7	**0.010**	1.00 (Ref.)
		AG/GG	517	98.1	502	95.3		**2.57 (1.22–5.41)**
	Alleles	A	195	18.5	225	21.3	0.102	1.00 (Ref.)
		G	859	81.5	829	78.7		1.20 (0.96–1.48)
DNMT1	rs2228611							
	Codominant	GG	261	49.5	263	49.9	**0.017**	1.00 (Ref.)
		AG	242	45.9	218	41.4		1.12 (0.87–1.44)
		AA	24	4.6	46	8.7		**0.53 (0.32–0.90)**
	Dominant	GG	261	49.5	263	49.9	0.902	1.00 (Ref.)
		AA/AG	266	50.5	264	50.1		1.02 (0.80–1.30)
	Recessive	AA	24	4.6	46	8.7	**0.007**	1.00 (Ref.)
		AG/GG	503	95.1	481	91.3		**1.98 (1.19–3.20)**
	Alleles	A	290	27.5	310	29.4	0.334	1.00 (Ref.)
		G	764	72.5	744	70.6		1.09 (0.90–1.32)

^a^Two-sided χ^2^ test.

^b^Adjusted for age, sex, smoking, drinking in logistic regression model.

### Stratified analysis of rs2802292, rs10457180, and rs12206094 polymorphisms and NIHL risk

The impacts of the rs7578575, rs749131, rs1550117, and rs2228611 genotypes on a series of NIHL risk characteristics were analyzed under a recessive model, and the results are shown in Table 4. We selected 16 years as work time standard as approximately half of the subjects were ≤6 work years, and the other were >16 work years. Significant differences between cases and controls were observed in the genotype distributions of rs2228611 in those with noise exposure time ≤16 years (OR = 4.05, 95% CI = 1.71–9.60) and in the genotype distributions of rs749131 in those with noise exposure time >16 years (OR = 2.34, 95% CI = 1.36–4.03). For persons exposed to ≤85 dB, those carrying the rs749131 GG (OR = 1.97, 95% CI = 1.03–3.84) or rs1550117 AG + GG genotypes (OR = 3.22, 95% CI = 1.02–10.16) exhibited an increased risk for NIHL. Workers exposed to >92 dB carrying the rs2228611 AG + GG genotypes (OR = 4.30, 95% CI = 1.58–11.70) also exhibited an increased risk for NIHL.

**Table 4:** Stratified analysis of SNPs in a recessive model.

SNPs	Group	Genotype	Work time with noise (years)		Expose level with noise (dB)		
			≤16	>16	≤85	85–92	>92
rs7578575	case	AA	13	23	21	6	9
		AT/TT	212	279	219	95	177
	control	AA	15	21	20	6	7
		AT/TT	227	264	212	85	194
	*P* ^a^		0.848	0.909	0.960	0.341	0.503
	Adjusted OR (95% CI)^b^		0.95 (0.44–2.05)	1.00 (0.54–1.86)	1.03 (0.54–1.96)	0.51 (0.17–1.54)	1.38 (0.50–3.79)
rs749131	case	GG	27	47	28	20	26
		GT/TT	198	255	212	81	160
	control	GG	22	21	15	11	17
		GT/TT	220	264	217	83	184
	*P* ^a^		0.305	**0.002**	**0.050**	0.122	.0.084
	Adjusted OR (95% CI)^b^		1.37 (0.75–2.50)	**2.34 (1.36–4.03)**	**1.97 (1.03–3.84)**	1.85 (0.82–4.17)	1.77 (0.92–3.38)
rs1550117	case	AG/GG	222	295	236	98	183
		AA	3	7	4	3	3
	control	AG/GG	232	270	220	91	191
		AA	10	15	12	3	10
	*P* ^a^		0.066	0.060	**0.035**	0.929	0.067
	Adjusted OR (95% CI)^b^		3.11 (0.84–11.48)	2.41 (0.96–6.03)	**3.22 (1.02–10.16)**	1.17 (0.23–6.02)	3.15 (0.85–11.65)
rs2228611	case	AG/GG	218	285	226	96	181
		AA	7	17	14	5	5
	control	AG/GG	216	265	214	87	180
		AA	26	20	18	7	21
	*P* ^a^		**0.001**	0.489	0.406	0.469	**0.002**
	Adjusted OR (95% CI)^b^		**4.05 (1.71–9.60)**	1.25 (0.64–2.47)	1.31 (0.63–2.73)	1.75(0.52–5.93)	**4.30 (1.58–11.70)**

^a^Two-sided χ^2^ test.

^b^Adjusted for age, sex, smoking, drinking in logistic regression model.

### Association of *DNMT1* and *DNMT3A* haplotypes with NIHL risk

The haplotype frequencies of the four SNPs were compared between NIHL cases and controls (Table 5). Ten common haplotypes (frequency > 2%) derived from the four SNPs accounting for 95% of the haplotype variation were selected, and the rest of the haplotypes were pooled into the mixed group. The haplotypes AGGG and TGGA (rs7578578-rs749131-rs1550117-rs2228611) were found to be associated with an increased risk for NIHL (OR = 1.346 and 1.557, respectively).

**Table 5:** Frequencies of inferred haplotypes among the cases and controls and their association with risk of NIHL.

Haplotypes^a^	Case		Control		*P* ^b^	OR (95% CI)	Global *P*^*c*^
	n	%	n	%			
AGGA	63.52	6.0	60.48	5.7	0.799	1.048 (0.729–1.508)	**0.041**
AGGG	133.38	12.7	102.05	9.7	**0.033**	**1.346 (1.024–1.770)**	
ATAG	23.89	2.3	18.03	1.7	0.369	1.326 (0.715–2.459)	
ATGG	34.7	3.3	46.01	4.4	0.191	0.742 (0.473–1.163)	
TGGA	67.71	6.4	44.33	4.2	**0.025**	**1.557 (1.055–2.298)**	
TGGG	121.16	11.5	116.97	11.1	0.802	1.035 (0.790–1.356)	
TTAA	36.34	3.4	51.35	4.9	0.097	0.694 (0.449–1.070)	
TTAG	123.69	11.7	116.97	11.1	0.709	0.951 (0.731–1.238)	
TTGA	102.9	9.8	130.39	12.4	0.051	0.762 (0.579–1.002)	
TTGG	317.97	30.2	322.23	30.6	0.784	0.974 (0.808–1.174)	
Others^d^	28.74	2.8	33.51	3.2		1.00 (Ref.)	

^a^The alleles of haplotypes were arrayed as rs7578578-rs749131-rs1550117-rs2228611.

^b^Two-sided χ^2^ test.

^c^Generated by permutation test with 1000 times of simulation.

^d^Haplotypes with a frequency <0.02 (AGAA/AGAG/ATAA/ATGA/ATGG/TGAA/TGAG) were pooled into the mixed group.

### Association between high-frequency hearing threshold shift and SNP genotypes

**Figure 1:**
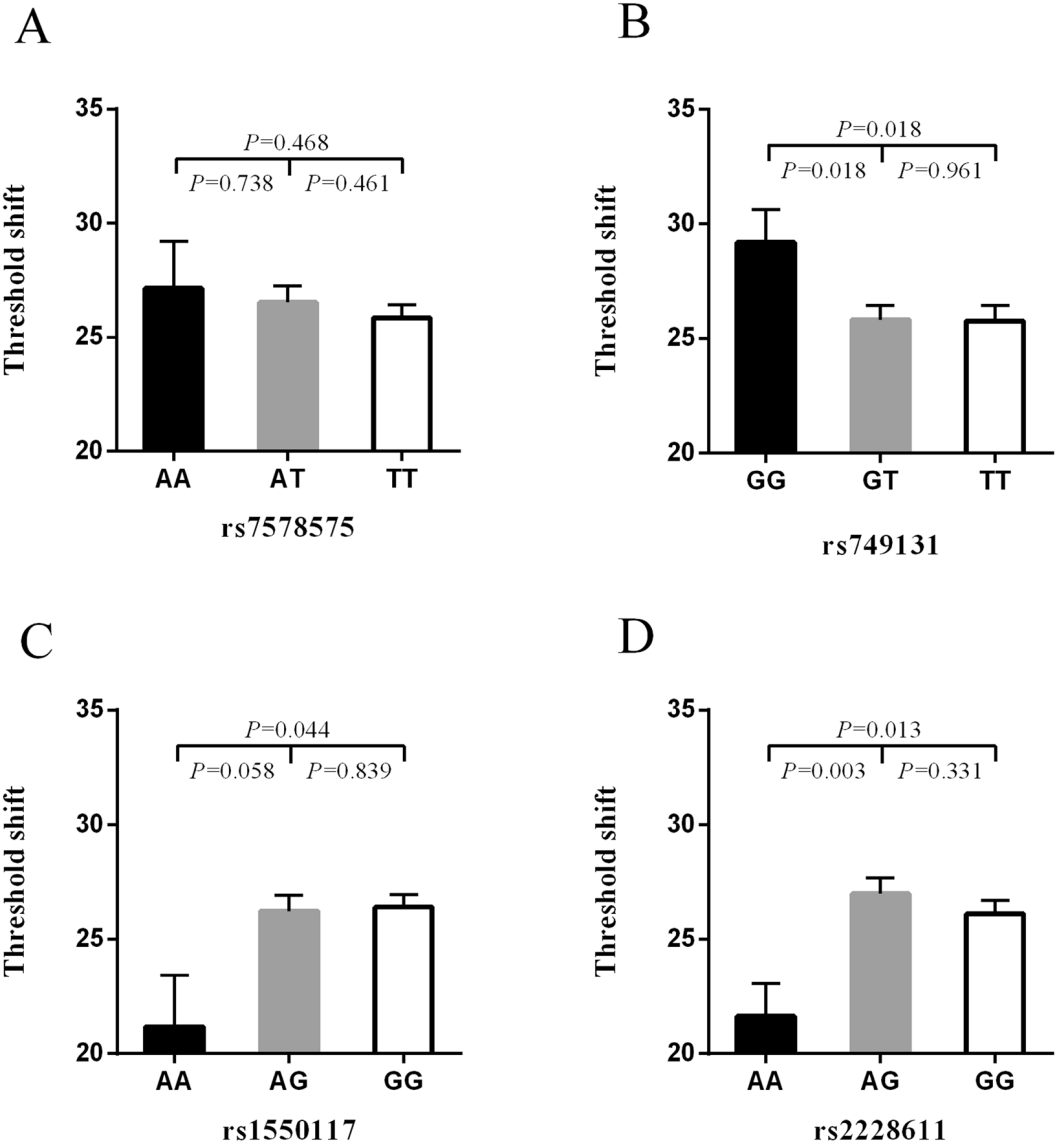
Comparison of high-frequency hearing threshold shift of all subjects. Comparison of high-frequency hearing threshold shift of rs7578575 (**A**), rs749131 (**B**), rs1550117 (**C**) and rs2228611 (**D**) genotypes in all subjects. Data are presented as mean ± SE and analyzed by ANOVA.

Figure 1 shows the results of the association analysis of the high-frequency hearing threshold shift and the genotypes at rs7578575, rs749131, rs1550117, and rs2228611 in the 1054 noise-exposed workers. Subjects with the GG genotype at rs749131 exhibited a significantly larger high-frequency hearing threshold shift than those with the GT or TT genotypes (*P* = 0.018). In addition, those with the GG genotype at rs1550117 and AG + GG genotypes at rs2228611 were found to have higher high-frequency hearing threshold shifts than those with the AA genotype.

### Multifactor dimensionality reduction analysis of interactions among SNPs

**Figure 2:**
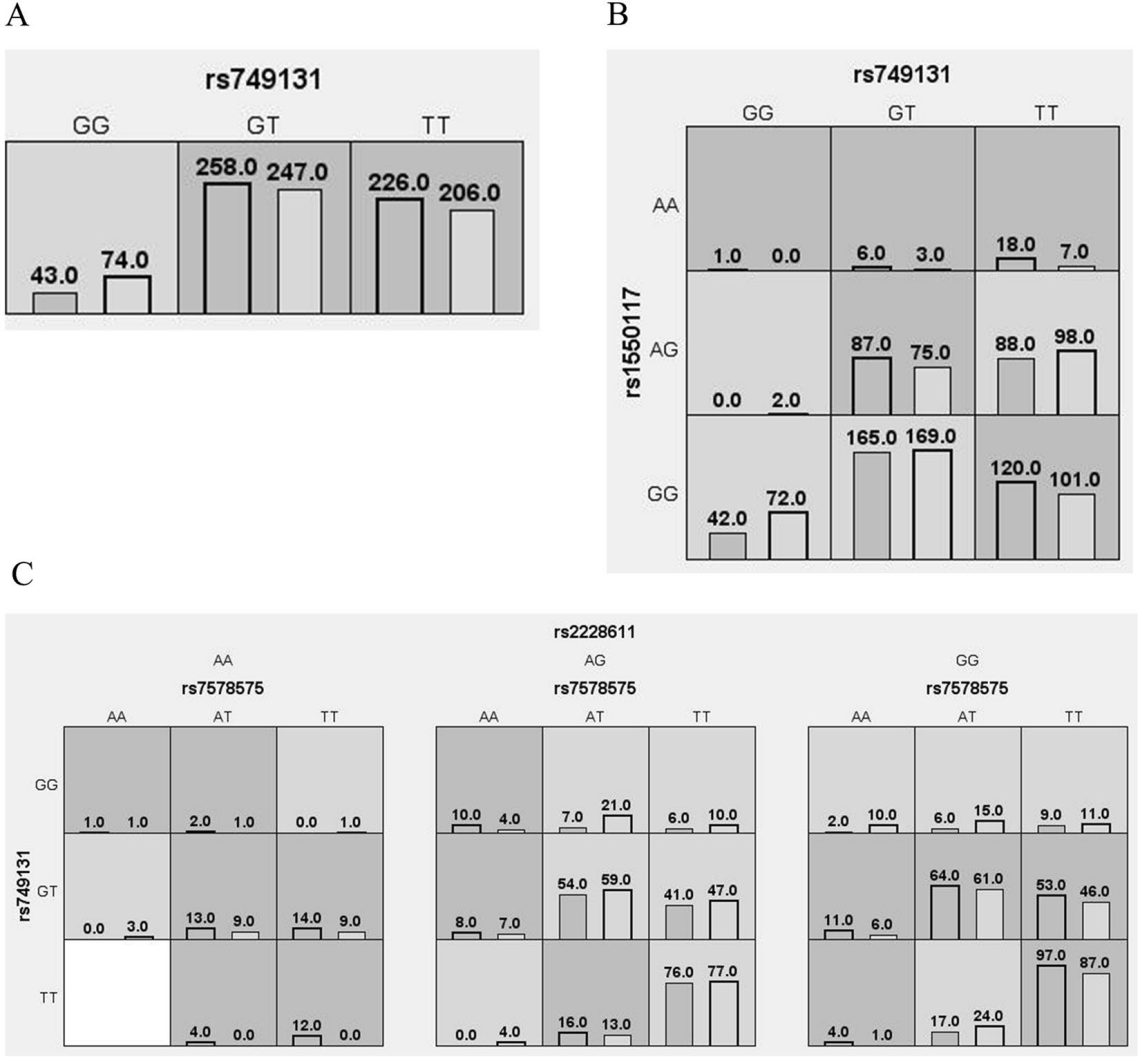
Graph model of the interaction between the four SNPs. Dark gray and light gray boxes presented the high- and low-risk factor combinations, respectively. Left bars within each box represented case while the right bars represented control. The heights of the bars are proportional to the sum of samples in each group.

The results of the multifactor dimensionality analysis of the interactions among the four SNPs are presented in Table 6 and Fig. 2. Figure 2 shows the distribution of high-risk and low-risk genotypes in the best locus model. The interaction results suggested that interactions between rs749131 and rs1550117 and among rs7578575, rs749131, and rs2228611 were associated with an increased NIHL risk (OR = 1.44 and 1.63, *P* = 0.0024 and *P* < 0.0001, respectively).

**Table 6:** MDR analysis results of the interaction between the four SNPs.

Model	Training balanced accuracy	Testing balanced accuracy	Cross-validation consistency	*P*	OR (95%CI)
rs749131	0.5299	0.5114	8/10	0.0024	1.84 (1.24–2.74)
rs749131-rs1550117	0.5465	0.5038	5/10	0.0038	1.44 (1.13–1.85)
rs7578575-rs749131-rs2228611	0.5650	0.5085	4/10	<0.0001	1.63 (1.28–2.08)

### Identification of promoter activity associated with SNP

**Figure 3:**
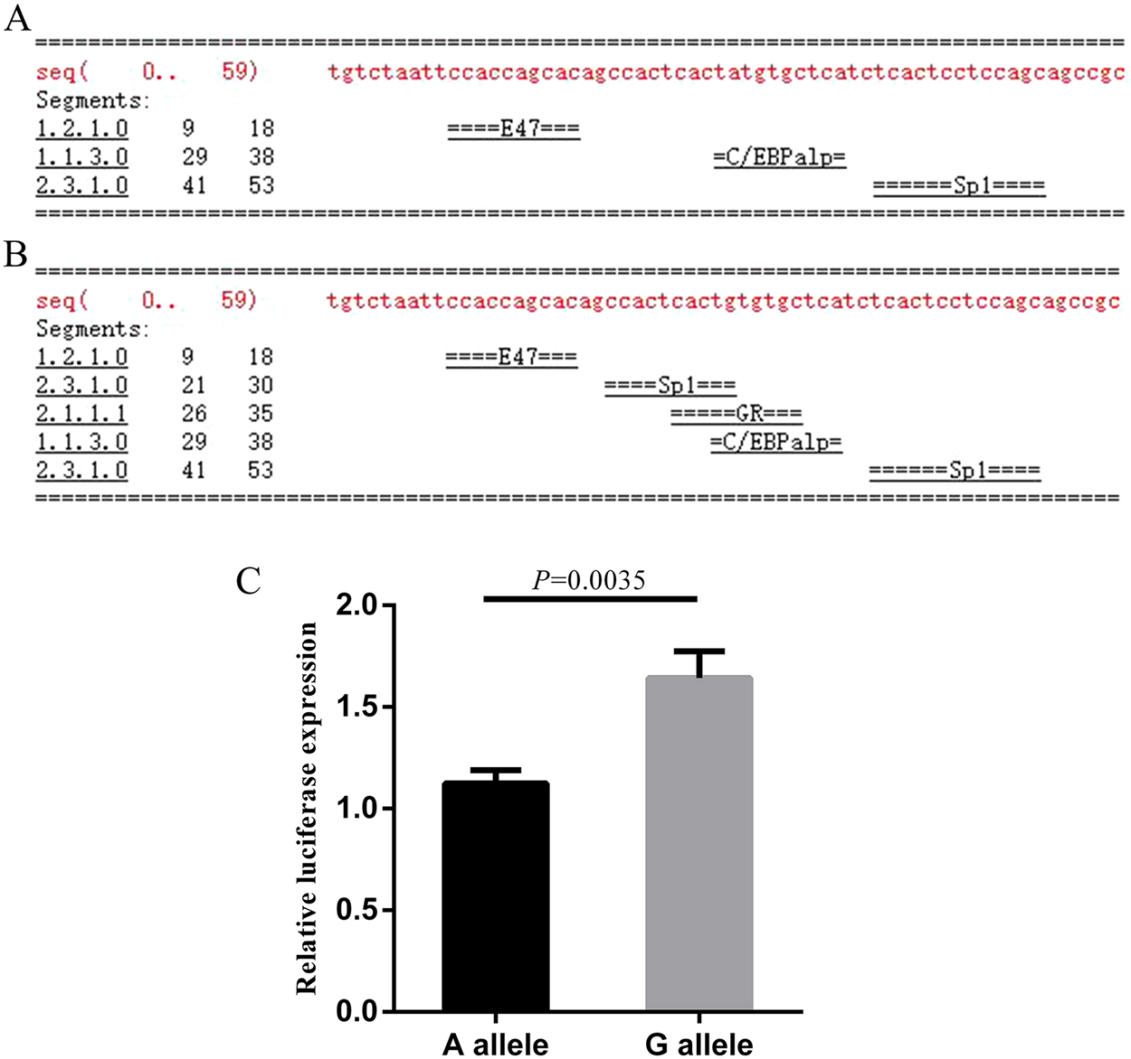
Identification of promoter activity in SNP rs1550117 region. Transcription factor binding sites prediction of intron 1 region containing rs1550117 G allele (**A**) and A allele (**B**). The arrow represents the transcription factor E47 binding site (CCACCAGCAC) from 9 to 18 bp. (**C**) Luciferase reporter assay of the luciferase activity of rs15550117.

Transcription factor binding site prediction by TRANSFAC showed that the version of *DNMT3A* intron 1 containing the rs1550117 G allele (Fig. 3A) may exhibit higher transcription factor binding activity than that containing the A allele (Fig. 3B). As shown in Fig. 3C, a subsequent luciferase reporter assay showed that the luciferase activity of intron 1 containing the G allele was significantly higher than that of intron 1 carrying the A allele in HEI-OC1 cells (*P *= 0.0035), indicating that there may be allele-specific modulation of *DNMT3A* expression, with the allele at rs1550117 potentially determining transcription factor binding affinity.

## Discussion

SNPs are a common type of genetic variation in the human genome, with about 15 million SNPs among all humans^23^. Analysis of haplotypes, defined as a specific set of alleles observed on a single chromosome or part of a chromosome, has been an integral part of human genetics for decades^24^. However, the genomic distribution of SNPs is not homogeneous, with most SNPs occurring in noncoding regions. At present, SNP detection methods include denaturing gradient gel electrophoresis (DGGE), single-strand conformational polymorphism (SSCP), cleaved amplified polymorphic sequence (CAPS), and allele-specific PCR (such as TaqMan genotype-PCR).

In the current study, a genetic association analysis was performed with four selected *DNMT1* and *DNMT3A* SNPs (rs7578575, rs749131, rs1550117, and rs2228611) in 527 NIHL patients and 527 controls using TaqMan genotyping. The results revealed that the rs749131 GG and rs1550117 AG/GG genotypes of *DNMT3A* and the rs2228611 AG/GG genotype of *DNMT1* are associated with a significantly higher NIHL risk. Subsequent haplotype analysis shows that the haplotypes AGGG and TGGA (rs7578578-rs749131-rs1550117-rs2228611) confer increased risk of NIHL. These findings support our hypothesis that *DNMT1* and *DNMT3A* polymorphisms may contribute to NIHL susceptibility in the Chinese population. To our knowledge, this is the first association study showing that *DNMT3A* genes are correlated with increased NIHL risk in the Chinese population.

To date, defects in DNA methylation have been identified as causative or contributing to numerous cancers, developmental syndromes, genetic disorders, and complex conditions^25^. *DNMT1* maintains methylation patterns, preserving epigenetic information across subsequent cellular generations, whereas *DNMT3A* establishes new patterns of methylation. Currently, numerous hearing impairment diseases have been shown to be caused by mutations in the maintenance methyltransferase DNMT1 and corresponding defects in the DNA methylation profiles of patients. In our study, mutation from A to G at SNP rs1550117 was significantly associated with increased NIHL risk. In addition, the G allele resulted in increased promoter activity of *DMNT3A* compared to the A allele. Despite the fact that rs749131 is located in a noncoding region of the *DNMT3A* gene, numerous studies have demonstrated functional consequences of noncoding SNPs involved in the regulation of protein-coding genes and long noncoding RNAs^26,27^. rs2228611 is a synonymous mutation located in the *DNMT1* gene. Kimchi-Sarfaty reported that a synonymous SNP in the *MDR1* gene, part of a haplotype previously linked to the altered function of the *MDR1* gene product P-glycoprotein (P-gp), nonetheless resulted in P-gp with altered drug and inhibitor interactions^28^. This demonstrates that naturally occurring synonymous SNPs can lead to the synthesis of protein products with the same amino acid sequence but different structural and functional properties. Supek *et al*. also reported that synonymous mutations can be oncogenic by altering transcript splicing, thereby affecting protein function^29^.

Our current study has several potential limitations. First, while the sample size of our study was relatively large compared to previous research, the power of the statistical tests may not be sufficient to detect the small biological effects of individual SNPs. Therefore, a larger sample size and cohort studies are needed in the future to confirm the effects of the *DNMT* polymorphisms on NIHL risk. Second, the subjects in this case-control study were limited to those in the Chinese population. Thus, our results may be more applicable to the Chinese Han population, with limited external generalizability. Third, rs749131 and rs2228611 are noncoding SNPs; thus, we assume that these variants are linked with one or more functional variants within the *DNMT* genes or their regulatory regions. Future fine mapping of these *DNMT* genes may detect such functional variants.

## Conclusions

In conclusion, genotypes at rs749131 G and rs1550117 G of *DNMT3A* and rs2228611 G of *DNMT1* are associated with a significantly higher risk of NIHL. Thus, our findings indicate that *DNMT* SNPs (rs749131, rs1550117, and rs2228611) may play crucial roles in NIHL and have the potential to serve as biomarkers for noise-exposed workers.
